# Supplementary figures and images for: Hemi- and Homozygous Loss-of-Function Mutations in DSG2 (Desmoglein-2) Cause Recessive Arrhythmogenic Cardiomyopathy with an Early Onset
Source: Int J Mol Sci. 2021 Apr 6;22(7):3786. doi: 10.3390/ijms22073786 (PMC8038858; doi:10.3390/ijms22073786)

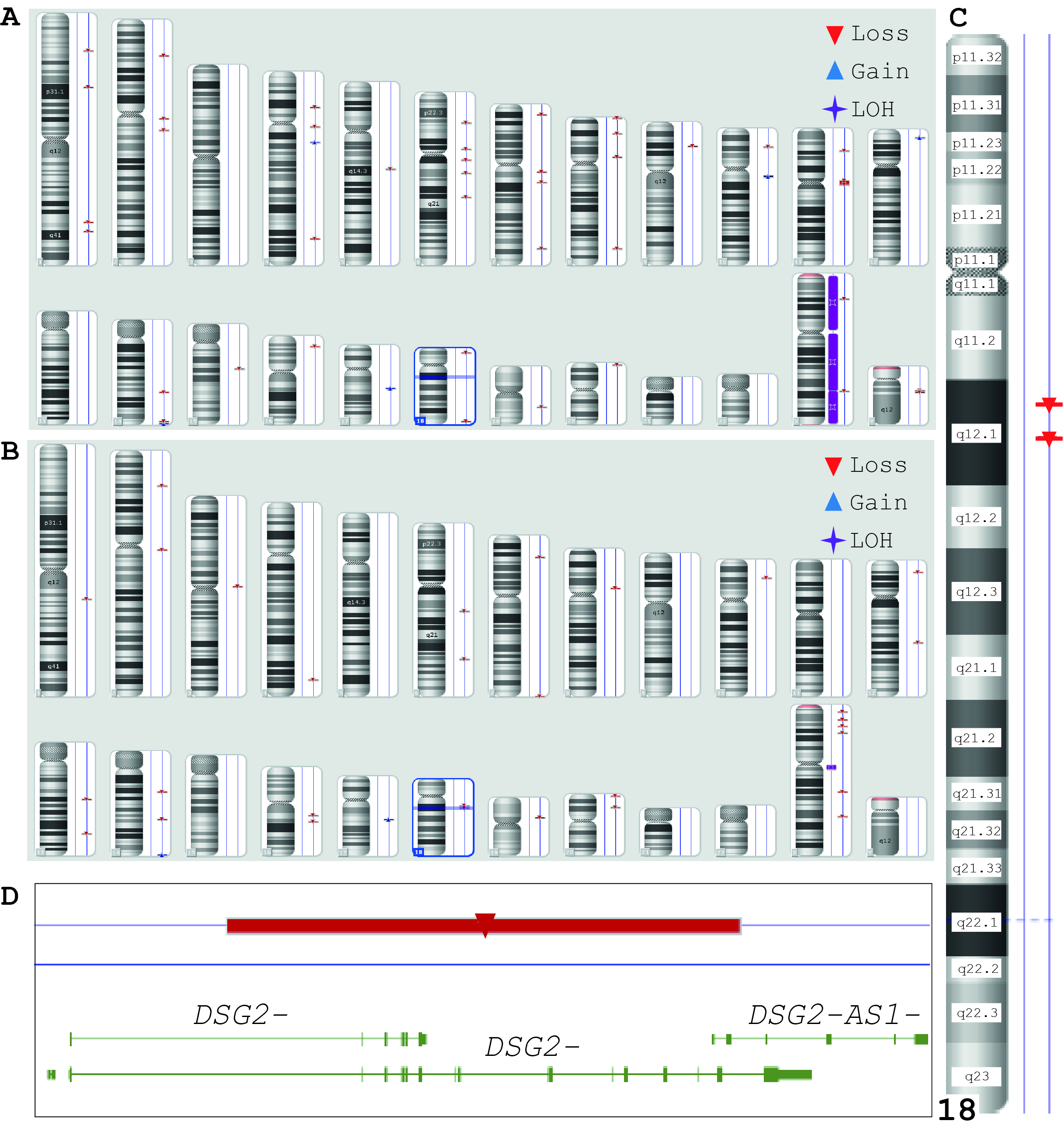

Supplement: Supplementary file 1 [file ijms-22-03786-s001.zip › ijms-1153246-supplementary/Supplementary Figure.tif]
